# Identification of GCK-MODY in cases of neonatal hyperglycemia: a case series and review of clinical features

**DOI:** 10.1111/pedi.13239

**Published:** 2021-06-10

**Authors:** Alice E Hughes, Elisa De Franco, Evgenia Globa, Nataliya Zelinska, Dörte Hilgard, Popi Sifianou, Andrew T Hattersley, Sarah E Flanagan

**Affiliations:** 1 Institute of Biomedical and Clinical Science, University of Exeter Medical School, Exeter, UK; 2 Department of Pediatric Endocrinology, Ukrainian Center of Endocrine Surgery, MoH of Ukraine, Kyiv, Ukraine; 3 Pediatric Practice, Pediatric Endocrinology and Diabetology, Witten, Germany; 4 Department of Neonatology, General Hospital “Elena Venizelou”, Athens, Greece

**Keywords:** Glucokinase, monogenic diabetes of the young, GCK-MODY, hyperglycemia, neonatal diabetes

## Abstract

Heterozygous mutations in *GCK* result in a persistent, mildly raised glucose from birth, but it is usually diagnosed in adulthood as maturity-onset diabetes of the young (MODY), where hyperglycemia is often an incidental finding. The hyperglycemia of GCK-MODY is benign and does not require treatment, but is important to be aware of, particularly in females where it has implications for managing pregnancy. We present three cases of neonatal hyperglycemia resulting from a heterozygous mutation in *GCK*, illustrating its clinical presentation and evolution in early life. In summary, as with adults, neonatal hyperglycemia is an incidental finding, does not require treatment and has no adverse consequences for health. Neonates and their parents should be referred for genetic testing to confirm the diagnosis, avoid a label of diabetes and enable pregnancy counselling for females found to be affected.

## Introduction

Heterozygous inactivating mutations in the gene encoding the glucose sensing enzyme glucokinase, *GCK*, are common (1 in 1,000 population prevalence^1^) and result in partial glucokinase deficiency^2^. This causes mild hyperglycemia (tightly regulated between 5.5-8 mmol/L^3^), often diagnosed incidentally in adulthood as maturity-onset diabetes of the young (MODY)^1,4^. The degree of hyperglycemia is uniform irrespective of mutation type^5^, and treatment of hyperglycemia is not required^6^, as it does not result in the micro- or macrovascular complications seen with type 1 and type 2 diabetes^7^. However, it does have implications for women of reproductive age, since fetuses who have not inherited the mutation from their mother secrete higher levels of insulin in response to maternal hyperglycemia, so are at risk of macrosomia and its associated pregnancy complications^8,9^. GCK-MODY is frequently diagnosed in pregnancy, either because of routine screening for gestational diabetes or a history of fetal macrosomia^10^.

Neonatal diabetes (NDM) is diagnosed in the first six months of life and affects approximately 1 in 100,000 live births^11^. It can be permanent (PNDM), requiring lifelong treatment, or transient (TNDM), where diabetes remits but typically returns in childhood or early adulthood^12^. The clinical presentation of NDM is usually severe, with marked hyperglycemia (frequently >30 mmol/L) and ketoacidosis^13^, and treatment is required to maintain euglycemia.

NDM may be isolated or diagnosed in association with other conditions or congenital anomalies. NDM has a known genetic etiology in almost 90% of cases, with a mutation in one of 28 known disease-causing genes affecting glucose metabolism, insulin secretion or pancreatic development^14–21^. Obtaining a genetic diagnosis is important, since prognosis and approach to monitoring and treatment are dependent on the underlying genetic cause^22–25^.

Bi-allelic inactivating mutations in *GCK* result in complete glucokinase deficiency and are an infrequent cause of PNDM, accounting for ~3% of cases with a confirmed genetic diagnosis^14^, but an important differential diagnosis of hyperglycemia in infants with very low birth weight (typically <-3 standard deviations below the mean for sex and gestational age due to substantially reduced fetal insulin-mediated growth)^26^. This contrasts with heterozygous inactivating mutations in *GCK* which cause GCK-MODY, where the degree of fetal insulin secretion and its effect on birth weight is determined by the parent-of-origin of the mutation^8^. Birth weight is reduced by approximately 500 g when the mother is unaffected and the fetus has inherited the mutation from the father, whereas birth weight is typically in the normal range when inherited from a mother with GCK-MODY^8,9^.

Family studies in GCK-MODY pedigrees^27^ and genetic screening for suspected GCK-MODY^28^ have shown that hyperglycemia is present from birth in affected individuals. However, it is rare for it to be recognised in neonates and considered as a cause of neonatal hyperglycemia in the absence of a known family history. Here, we present three cases of neonatal hyperglycemia who did not have a family history of GCK-MODY and were referred for genetic testing for NDM. We describe how GCK-MODY can present in newborns and emphasise the importance of considering it in cases of asymptomatic, mild neonatal hyperglycemia not requiring treatment.

## Methods

Out of 2,203 individuals identified as having hyperglycemia in the first six months of life and referred to Exeter Genomics Laboratory for genetic testing of NDM, we identified three individuals with heterozygous inactivating mutations in *GCK* (NM_000162.3). The *GCK* mutations were identified either by PCR amplification followed by Sanger sequencing (primers available on request) or by analysis of all the known NDM genes using an in-house targeted next generation sequencing (tNGS) assay as previously described^29^. Variant classification was performed according to the ACMG international guidelines for variant interpretation^30^. There was no known history of a genetic cause for diabetes in their families. Their parents were genotyped once a mutation in *GCK* was identified in the child.

Clinical data (Table 1) was collected from the cases’ health records. Birth weight standard deviation scores (SDS) were calculated using the INTERGROWTH-21^st^ standards^31^ and most recent available weight and height SDS were calculated using the WHO Child Growth Standards or Reference^32,33^. Informed consent was received from their parents and ethics approval for research was provided by the North Wales Research Ethics Committee.

## Cases

### Case 1

**Figure 1:**
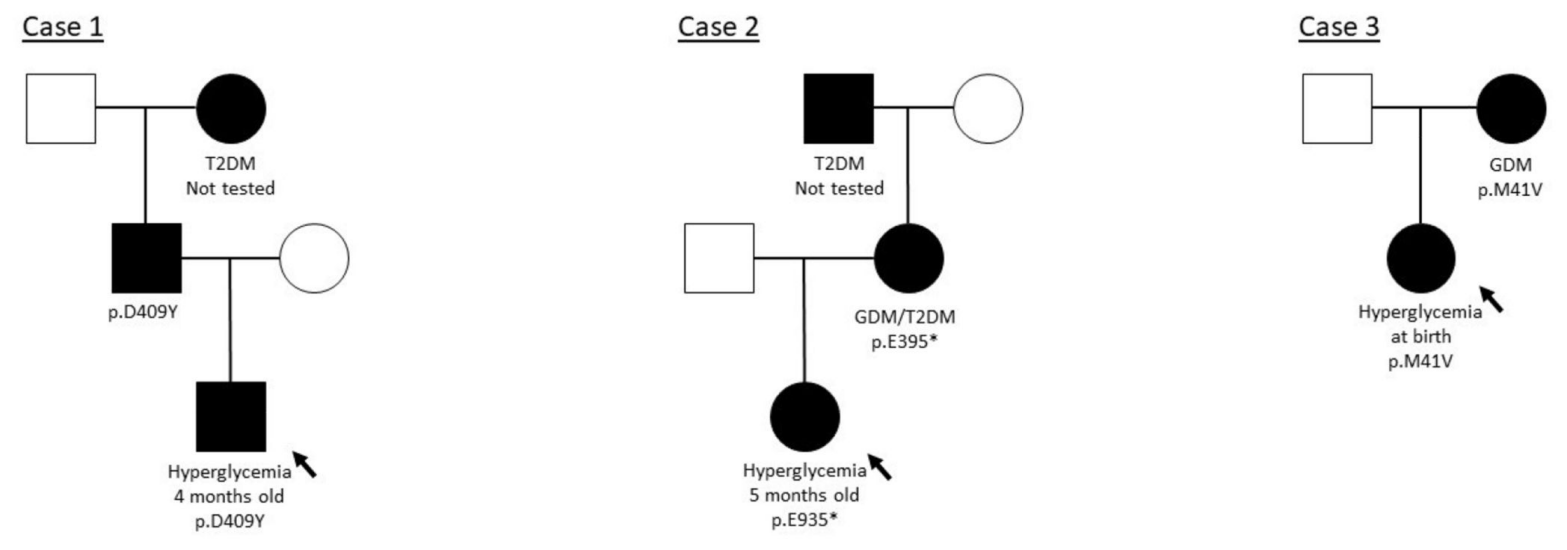
Partial pedigrees for cases of GCK-MODY diagnosed where hyperglycemia was first identified in the neonatal period. Filled symbols represent individuals with a genetic diagnosis of GCK-MODY, genotypes are provided under the symbols. The clinical diagnosis is provided under the symbols for individuals affected by diabetes (for the father of Case 1, the affected phenotype is assumed based on genotype). GDM = Gestational Diabetes Mellitus. T2D = Type 2 diabetes. An arrow points to the proband in each family.

A healthy male infant of Northern European ancestry was born at 39 weeks gestation to non-consanguineous parents weighing 3.9 kg (1.51 SDS). Pregnancy and delivery were uneventful, but he was admitted to hospital with signs suggestive of bowel obstruction at four months of age. He was diagnosed with Hirschsprung’s disease and underwent surgery. His blood glucose prior to surgery was 8 mmol/L and varied between 11 and 16 mmol/L during surgery. He did not require insulin treatment. At six months of age his case was reviewed by a Pediatric Endocrinologist who established that his paternal grandmother had a history of type 2 diabetes but there was no other significant family history. His HbA1c was 47.5 mmol/mol, C-peptide was 190 pmol/L and an autoantibody screen was negative. He was referred for genetic testing for NDM. He first underwent Sanger sequencing of the *ABCC8*, *KCNJ11* and *INS* genes and was screened for a methylation defect at the 6q24 locus. The results of these tests were negative, so targeted next generation sequencing (tNGS) of a panel of 28 known NDM genes^29^ was performed and a heterozygous missense variant in *GCK* (c.1225G>T (p.D409Y)) was identified (Figure 1). This variant was classified as likely pathogenic. The proband’s father, who was not known to have diabetes, was heterozygous for the same *GCK* mutation. A sample from the maternal grandmother was not available for testing. The proband’s most recent clinical assessment at 6 years of age found him to be fit and well and his hyperglycemia remained untreated.

### Case 2

A healthy female infant of Eastern European ancestry was born at 37 weeks gestation to non-consanguineous parents weighing 3.1 kg (0.77 SDS). Pregnancy and delivery were uneventful. The girl’s blood glucose was measured during a viral illness at four months of age and found to be raised at 6 mmol/L. No treatment was given and her blood glucose was tested intermittently in childhood (7.4 mmol/L at eight months old, 6 mmol/L at three years old and 5.9 mmol/L at four years old) before a specialist review at six years and seven months of age. She was of a normal height and weight (1.6 SDS and 1.4 SDS, respectively), but was noted to have some signs of early pubarche (Tanner Stage 2). At the time her HbA1c was 49.7 mmol/mol, serum C-peptide was 255 pmol/L and an autoantibody screen, including GAD, IA2 and ICA, was negative. Her mother had been diagnosed with gestational diabetes (GDM) in a previous pregnancy at 19 years old and was subsequently diagnosed with type 2 diabetes aged 23 years. The mother was treated with a low carbohydrate diet (including in her pregnancy with the proband) until the age of 26 years, when she was started on metformin and saxagliptin. In addition to her mother’s history of diabetes, the girl’s maternal grandfather had been diagnosed with type 2 diabetes aged 46 years. In view of the history, Sanger sequencing of *GCK* was performed, which revealed a heterozygous *GCK* nonsense mutation (c.1183G>T (p.E395*)), which was also present in her mother (Figure 1). A sample from the maternal grandfather was not available for testing. The girl’s most recent HbA1c at the age of 11 years was 38.7 mmol/mol and she was known to be fit and well without treatment of hyperglycemia at 12 years of age. This case has been described briefly in the literature^34^.

### Case 3

A female infant of Greek ancestry was born at 38 weeks gestation to non-consanguineous parents weighing 2.6 kg (−0.80 SDS). Her mother was treated for GDM with insulin in pregnancy. The infant’s blood glucose was measured as a routine screen in the first 24 hours of life and was found to be high; pre-feed blood glucose levels ranged between 5.5 and 10 mmol/L and the highest measured was 12.6 mmol/L. There was no ketonuria and her serum C-peptide was 96 pmol/L pre-feed and 536 pmol/L post-feed. She was noted to have macroglossia, but was otherwise well and her hyperglycemia did not require treatment. She was referred for genetic testing for NDM and underwent screening of the known genes by tNGS and methylation-specific MS-MLPA of the 6q24 locus. A heterozygous novel missense variant was identified in *GCK* (c.121A>G (p.M41V)) and 6q24 MS-MLPA was negative. The *GCK* variant was classified as likely pathogenic. The proband had inherited this mutation from her mother, confirming a diagnosis of GCK-MODY in both. At her most recent assessment at the age of 15 months, her hyperglycemia remained untreated and she was well (Table 1).

## Discussion

In this series of GCK-MODY diagnosed in cases referred for genetic testing for suspected NDM, we have shown that neonatal hyperglycemia is an incidental finding, does not require treatment and follows a benign course in childhood. Retrospective studies in individuals with a known family history or clinically suspected GCK-MODY have previously confirmed that hyperglycemia can occur from birth^27,28^. Previous series where genetic screening was performed in individuals with incidental hyperglycemia have identified children who presented as young as one year of age^35,36^. However, referral for genetic testing with suspected NDM is rare, and these cases confirm the importance of considering GCK-MODY as a cause of neonatal hyperglycaemia.

In pregnancies affected by GCK-MODY, as fetal growth depends on the parent-of-origin of the mutation, birth weight combined with parental history can provide useful clues to the underlying diagnosis in an otherwise well infant with hyperglycemia. However, birth weight is not always a reliable indicator, as fetal insulin- and non-insulin-mediated growth will have different effects between different individuals. In Case 3, the infant had a birth weight lower than might be expected for a mother who had GDM requiring insulin. It is possible that maternal insulin treatment contributed to the lower birth weight in this case^37^. The birth weight for the infant in Case 2 was normal, despite her mother’s history of diabetes diagnosed after screening in a previous pregnancy. Therefore, for a hyperglycemic infant of normal or low birth weight born to a mother with GDM, particularly where maternal hyperglycemia has failed to respond to insulin treatment in pregnancy^9,38^ and persists postpartum, GCK-MODY is an important consideration. Neonatal hypoglycemia and macrosomia may be evident in the infant who has not inherited the same *GCK* mutation as their mother^9^, so a high birth weight and history of neonatal hypoglycemia in siblings may also provide a useful clue to the diagnosis. Where the mutation is inherited from the father, birth weight is reduced by approximately 500 g on average^8^, but this was not seen in Case 1, and his father (who he had inherited the *GCK* mutation from) was not known to have diabetes. It is very common for adult males to be unaware of their diagnosis, since the hyperglycemia of GCK-MODY does not cause symptoms or complications, so it may be less clear-cut when the mutation is inherited from the father. However, where there is a strong family history of “type 2 diabetes” without other clinical features or complications and diagnosed at a young age (as was seen in Case 2), this should also raise suspicion for GCK-MODY.

We found that GCK-MODY was a rare diagnosis for infants with neonatal hyperglycemia and without a known family history referred for genetic testing (~0.1%). These three cases also had other clinical conditions or signs which might have prompted the clinicians to consider an underlying genetic aetiology. Hirschsprung’s disease and possibly precocious puberty were present in Case 1 and 2, respectively, but these are not related to hyperglycemia and have not been reported to be typical clinical features for individuals with NDM. The infant in Case 3 had macroglossia, which has been identified as a feature of some cases of 6q24 TNDM^39,40^, but this is a variable and imprecise sign which has no known relation to GCK-MODY. Overall, the presence of other features emphasises that these children are at risk of other pediatric conditions and these should not be attributed to GCK-MODY.

Hyperglycemia is stable in GCK-MODY, as shown by Case 2 where her glucose was maintained within a tight range when tested at different points in early childhood, and no progression in HbA1c levels in the absence of treatment in Cases 1 and 2. This is consistent with the adult literature, where HbA1c is relatively stable, showing a slight rise in later life which is also seen in individuals without diabetes^3^. Recent measures of height and weight in the cases reported here were also within the normal range, consistent with that seen in adults with GCK-MODY^41^.

In summary, although GCK-MODY is more commonly identified in adults as an incidental finding of hyperglycemia^1^, it can be identified at birth. As blood glucose monitoring becomes more frequent, and if neonatal bloodspot glucose becomes routine^42^, it may be diagnosed more often in the future. The distinguishing features are a mild hyperglycemia that does not require treatment or cause illness, unlike that seen in other cases of NDM, including complete glucokinase deficiency caused by bi-allelic inactivating mutations. Furthermore, the combination of hyperglycemia with a birth weight within the normal range, particularly where their mother also had GDM, and a strong family history of early-onset, non-progressive “type 2 diabetes” should raise suspicion for GCK-MODY. Genetic diagnosis is useful, since unnecessary treatment and monitoring are avoided. Additionally, for female infants and mothers of probands found to have the mutation, it allows preparation for future pregnancies, where antenatal management differs to typical cases of diabetes in pregnancy^1,9^.

## Figures and Tables

**Table 1:** Clinical characteristics of cases of neonatal hyperglycemia secondary to heterozygous mutations in *GCK*.

	Case 1	Case 2	Case 3
**Sex**	Male	Female	Female
**Country of origin**	Germany	Ukraine	Greece
**Ethnic ancestry**	Northern European	Eastern European	Greek
**Gestational age at birth**	39 weeks	37 weeks	38 weeks
**Birth weight and SDS for gestational age and sex** ^a^	3.9 kg (1.51 SDS)	3.1 kg (0.77 SDS)	2.6 kg (−0.80 SDS)
**Blood glucose at presentation**	8 mmol/L	6 mmol/L	5.5 mmol/L
**Age hyperglycaemia first identified**	4 months	5 months	24 hours
**Age of referral for genetic testing**	6 months	6 years and 7 months	1 month
**HbA1c at referral**	47.5 mmol/mol	49.7 mmol/mol	-
**C-peptide at referral**	190 pmol/L	257 pmol/L	536 pmol/L
**Autoantibody screen at referral**	Negative	Negative	-
**Treatment history**	Nil	Nil	Nil
**Most recent height and SDS for age and sex** ^b^	1.16 m (0.11 SDS)	1.27 m (1.6 SDS)	76 cm (−1.31 SDS)
**Most recent weight and SDS for age and sex** ^b^	22.5 kg (0.03 SDS)	27.0 kg (1.4 SDS)	10.2 kg (0.13 SDS)
**Most recent HbA1c (age measured)**	43.2 mmol/mol (6 years old)	38.7 mmol/mol (11 years old)	-

*GCK*, glucokinase gene; GDM, gestational diabetes; MODY, maturity-onset diabetes of the young; SDS, standard deviation score; T2DM, type 2 diabetes

a Calculated using the INTERGROWTH-21^st^ standards.^31^

b Calculated using the WHO Growth Standards (0-5 years) and Reference (6-19 years).^32,33^

## Data Availability

The data that supports this work is not freely available due to its identifiable nature, but reasonable requests for additional data can be made to the corresponding author.
